# Morning vs. afternoon saliva: limited diurnal variation in ion composition of unstimulated whole saliva

**DOI:** 10.1007/s00784-026-06797-9

**Published:** 2026-03-04

**Authors:** Joanita S. van Santen, Zainab Assy, Marwa Shanor, Maryam Kazemi, Kamran Nazmi, Henderikus Pots, Sarah Pringle, Floris J. Bikker

**Affiliations:** 1 Department of Oral Biochemistry, Academic Centre for Dentistry Amsterdam, University of Amsterdam and Vrije Universiteit Amsterdam, Gustav Mahlerlaan 3004, Amsterdam, 1081 LA the Netherlands; 2 Department of Rheumatology and Clinical Immunology, University Medical Centre Groningen, University of Groningen, Hanzeplein 1, Groningen, 9713 GZ the Netherlands

**Keywords:** Saliva, Circadian rhythm, Ions, Electrolytes, Diagnostics

## Abstract

**Objectives:**

Saliva is increasingly recognized as a valuable diagnostic fluid due to its non-invasive collection and wide range of biochemical markers. However, the potential influence of the circadian rhythm on salivary composition remains a concern, e.g. in clinical diagnostics. This study aimed to determine whether the time of collection—morning (9:00–11:00) versus afternoon (13:00–15:00)—significantly affects ion concentrations in unstimulated whole saliva (UWS).

**Materials and methods:**

Saliva samples from 30 healthy adults were analyzed for flow rate. Next, cortisol (as a reference marker for the circadian rhythm) and nine salivary ions were quantified using ELISA and capillary electrophoresis, respectively.

**Results:**

No significant differences were found in flow rate or cortisol levels between the two timepoints. Most ion concentrations, namely sodium, potassium, chloride, phosphate, sulphate, ammonium, and nitrate, also remained stable. In contrast, calcium and magnesium showed significant, opposing shifts: calcium was higher in the afternoon, while magnesium was higher in the morning.

**Conclusion:**

These findings suggest that, with some exceptions, salivary ion concentrations are largely unaffected by collection time.

**Clinical relevance:**

This supports the flexible use of UWS for diagnostic purposes without strict timing constraints, increasing its applicability in clinical and research settings.

## Introduction

Saliva has been gaining interest as a bodily fluid for diagnostic purposes, owing to the non-invasive nature of saliva sample collection and ease of storage [1]. For example, salivary ions have been extensively studied for their potential to aid in the diagnosis of Sjögren’s disease [2]. However, a key challenge in measuring salivary biomarkers for diagnostic purposes throughout the day is the potential influence of the circadian rhythm, proposed to lead to a reduced saliva production early in the morning and late evening, and subsequently the concentration of its components [3]. Thus, it is often suggested that it is best to collect saliva samples within a narrow time frame, to limit diurnal variation and subsequently boost reproducibility. However, there is currently no evidence that circadian variation significantly affects salivary ion concentrations when samples are collected during typical hospital working hours (e.g., between 9:00 and 17:00). With this study, we aim to elucidate whether the timing of saliva collection (morning or afternoon) has an impact on the measured salivary ion concentrations (sodium, potassium, chloride, phosphate, sulphate, ammonium, nitrate, magnesium and calcium) in unstimulated whole saliva (UWS) samples.

## Methods

The study received approval from the Ethical Examination Committee of the Academic Centre for Dentistry Amsterdam (ACTA) (protocol number: OBC-2023). The inclusion criteria were adults over the age of 18 years old. Exclusion criteria were systemic diseases and the use of xerogenic medication. The healthy participants were recruited amongst students and employees of the ACTA, between February 2023 and March 2024. Prior to participation, all volunteers received an information letter and signed an informed consent form. A total of 30 volunteers were recruited at the ACTA. The average age was 34,1 years old (range = 20–65 years), and 57% of all participants were female.

Participants were instructed to not eat, drink or practice oral hygiene one hour before the saliva collection moments. UWS was collected twice on the same day for each participant: once in the morning between 9:00 and 11:00 h and once in the afternoon between 13:00 and 15:00 h. For the saliva collection, participants were instructed to swallow one last time and then sit straight and relaxed, while letting the saliva pool in their mouth for 5 min. After the 5 min the collected saliva was drooled into a polypropylene-cup (Greiner Bio-One International GmbH, Alphen aan den Rijn, the Netherlands). If the participants could not manage to hold the saliva in their mouth for 5 min, they were allowed to expectorate the saliva into the cup in between as well. Saliva samples were centrifuged at 20,018 × *g* for 5 min at room temperature (RT), followed by aliquoting and storing of the supernatant at -20℃.

The salivary ion concentrations were determined making use of capillary electrophoresis (CAPEL-205; Lumex Instruments, Canada) equipped with a cassette containing a 60 cm capillary with an inner diameter of 75 μm (BGB Analytik Benelux B.V., Harderwijk, the Netherlands). The stored supernatants were thawed and again centrifuged at 20,018 ×*g* for 5 min at RT to remove any potential remaining debris. Then, 50 µL of the supernatant was diluted in 450 µL of MiliQ water. For cation and anion measurements, their respective manufacturers protocols were followed. For cations, the analysis time was extended to 7 min. The resulting electropherograms were interpreted making use of the accompanying Elforun-205 software (Envico, Zoeterwoude, the Netherlands). Ion concentrations were denoted in mM. All samples were analyzed in duplicate.

To measure the cortisol concentration in the UWS samples, a salivary cortisol ELISA kit (DRG Instruments GmbH, Marburg, Germany) was used. The measurements were executed according to the manufacturers protocol. The absorbance was determined making use of a Multiskan FC microplate photometer (Thermo Fisher Scientific Inc., Massachusetts, USA). Cortisol concentrations were denoted in ng/mL.

To determine if there were any differences for each ion between the morning samples and the afternoon samples, a Friedman test was performed, followed by paired Wilcoxon signed-ranks tests. The analyses and the creation of figures were both executed making use of Graphpad Prism v.10.4.1 (For Windows; Boston, Massachusetts, USA). Moreover, differences between men and women were assessed for the salivary FR, cortisol concentrations and each ion with a Mann Whitney U test, performed in IBM SPSS Statistics software v. 29.0.1.0 [4].

## Results

**Fig. 1:**
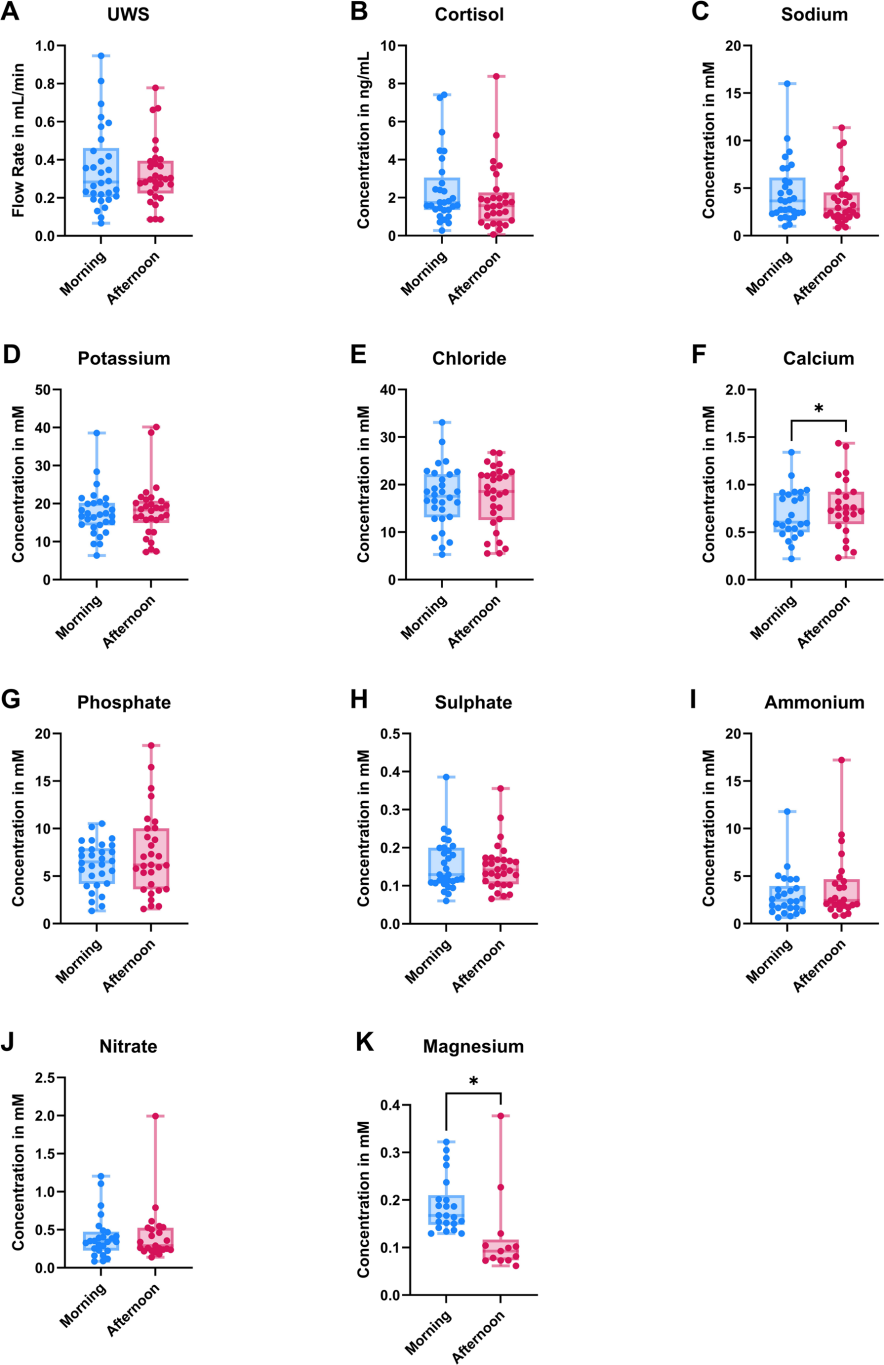
Box plots depicting **A** the median (and IQR) of the flow rate (in mL/min) of the unstimulated whole saliva (UWS) collected in the morning and the afternoon; **B** the median (and IQR) of the measured cortisol concentrations (in ng/mL) in the UWS samples from the morning and the afternoon; and the median (and IQR) of the concentrations (in mM) of **C** sodium, **D** potassium, **E** chloride, **F** calcium, **G** phosphate, **H** sulphate, **I** ammonium, **J** nitrate and **K** magnesium in the unstimulated whole saliva samples collected in the morning and afternoon. The error bars depict the minimum and maximum datapoint of the data shown. * *p* < 0.05

The median salivary flow rate (FR) of the collected UWS in the morning and the afternoon were 0,28 mL/min (IQR = 0,20 − 0,46 mL/min) and 0,30 mL/min (IQR = 0,22 − 0,39 mL/min) respectively (Fig. 1A). No significant differences were found between the UWS FR in the morning compared to the afternoon. Moreover, no differences were found between the cortisol concentrations of the UWS collected in the morning (median = 1,75 ng/mL, IQR = 1,35 − 3,06 ng/mL) compared to the afternoon (median = 1,58 ng/mL, IQR = 0,75 − 2,27 ng/mL) (Fig. 1B). Additionally, there were no significant differences between men and women for the UWS FR and cortisol results.

**Fig. 2:**
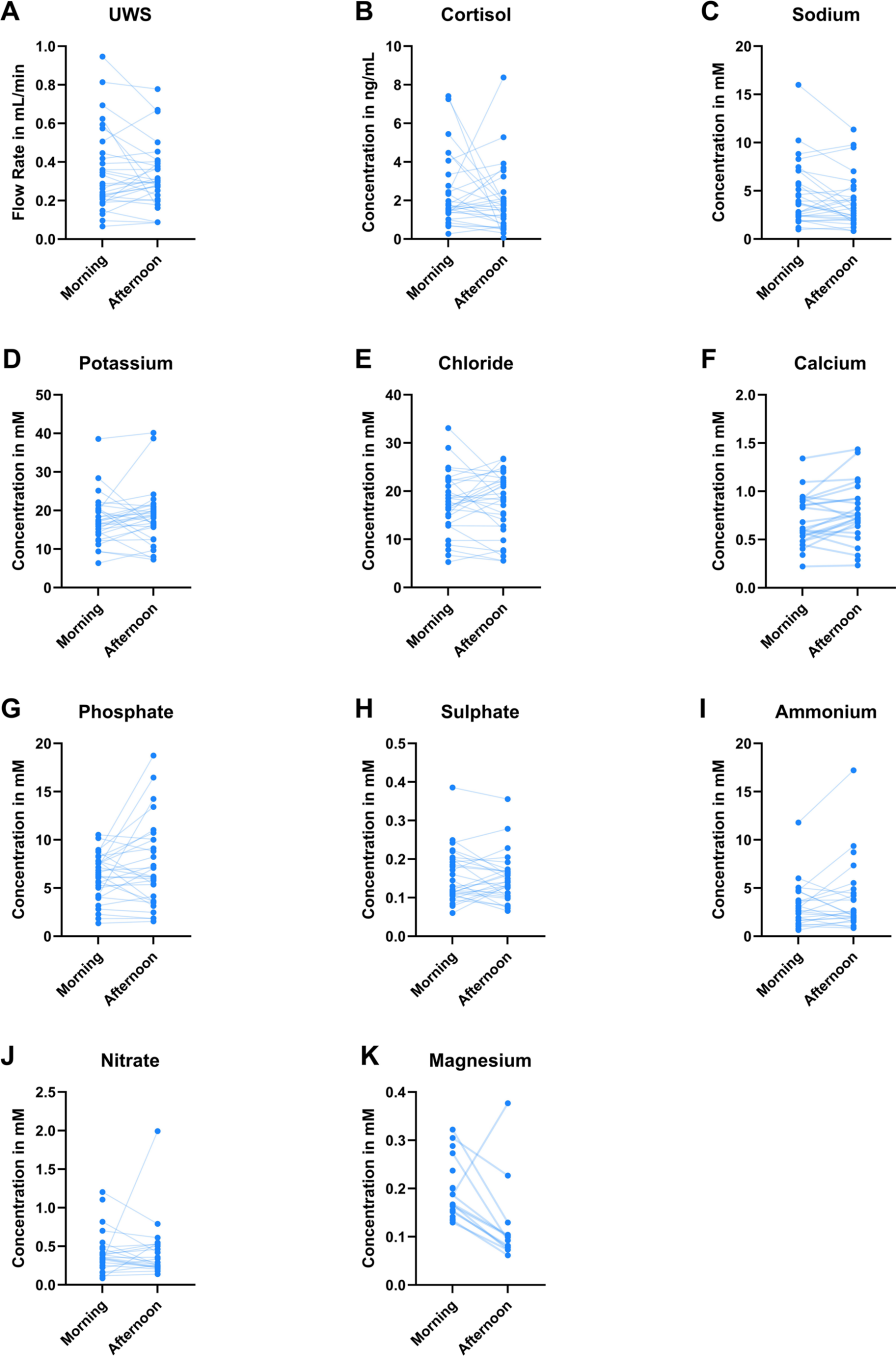
Line plots depicting the paired data points for **A** the unstimulated whole salivary flow rate (in mL/min) collected in the morning and the afternoon; **B** the cortisol concentrations (in ng/mL) in the UWS samples from the morning and the afternoon; and the concentrations in (mM) of **C** sodium, **D** potassium, **E** chloride, **F** calcium, **G** phosphate, **H** sulphate, **I** ammonium, **J** nitrate and **K** magnesium in the unstimulated whole saliva samples collected in the morning and afternoon

The ion concentration of sodium, potassium, chloride, calcium, phosphate, sulphate, ammonium, nitrate, and magnesium were determined (Fig. 1C–K). The only ions depicting a significant difference in concentration were calcium and magnesium. The calcium concentration in the afternoon was higher (median = 0,74 mM, IQR = 0,59 − 0,93 mM) than in the morning (median = 0,61 mM, IQR = 0,50 − 0,91 mM; *p* = 0,030; Fig. 1D). The magnesium concentration on the other hand, was higher in the morning (median = 0,17 mM, IQR = 0,15 − 0,21 mM), compared the afternoon (median = 0,09 mM, IQR = 0,07 − 0,12 mM; *p* = 0,027; Fig. 1K). Some data points are missing for magnesium, calcium and nitrate, due to those measurements falling below the detection limit of the CE system. In Fig. 2, the paired datapoints between the different collection times are connected to show the changes in UWS FR, cortisol and ion concentration for each individual between the morning and afternoon. It can be observed that the differences in results between morning and afternoon were not consistent between individuals for UWS, cortisol and for the ions. Moreover, if a person depicted a change in one of the variables, it did not perse correlate to a change in the same direction in one of the other variables. Lastly, no significant differences were found between men and women for any of the ions in the morning or afternoon.

## Discussion

The study aimed to investigate whether the collection time of saliva influences the ion concentrations measured in the saliva samples (i.e. the circadian rhythm), specifically for samples taken in the morning and the afternoon.

Our analysis showed that the cortisol concentrations – a previously established ‘reference marker’ of circadian rhythm – detected were not statistically different in the morning versus afternoon. Literature shows that there is a spike in cortisol secretion around the time of awakening, followed by a decrease of cortisol concentration during the day as per the circadian rhythm. This is, however, not the only moment of cortisol secretion throughout the day [5]. Cortisol is also secreted in pulsatile manner with intervals of approximately 90 min by the adrenal gland, following an ultradian rhythm [6–8]. Furthermore, secretion of cortisol has also been suggested to increase after meal consumption [9–12]. This may explain why cortisol concentration in the morning and afternoon saliva samples were comparable.

The measured UWS FR was comparable in the morning and the afternoon, the range which was furthermore in line with healthy reference values [13, 14]. There were also no significant differences in concentrations measured between the morning and afternoon for most of the salivary ions. Statistically significant differences were only observed in calcium and magnesium concentrations between the morning and afternoon UWS samples. Markedly, these differences were observed in opposing directions, with calcium increasing in the afternoon and magnesium decreasing. These observations might be related to one another. Literature suggests magnesium might play a regulatory role for calcium secretion in the acinar cells of the pancreas, where a decrease in magnesium leads to an increase in calcium and vice versa [15, 16]. Interestingly, there are many parallels to be drawn between the salivary glands and the pancreas [17, 18]. This leads to a potential hypothesis that magnesium might also operate as a regulator for calcium in the salivary glands. However, this does not yet explain the differences in salivary magnesium and calcium between the morning and afternoon. The potential relation between calcium and magnesium, and why they depict differences between the UWS collected in the morning and the afternoon remains to be elucidated.

A potential limitation of this study is that we only analyzed UWS, and not stimulated whole saliva as well. However, a previously published systematic review, indicated that using UWS for salivary ion measurements likely leads to less variation in the results and thus more reliability [2]. Another limitation could be that we did not assess the oral health status of the participants. This is something that could be of interest for a future follow-up.

## Conclusion

The present study demonstrates a comparison between UWS collected in the morning (9:00–11:00) and the afternoon (13:00–15:00), specifically focused on the measurement of salivary ion concentrations. No differences were observed in UWS FR, cortisol concentrations, and most salivary ions measured. Only magnesium and calcium showed differences between the two collection times. Depending on the salivary ion of interest, the collection time will not have to be considered when analyzing the obtained results. This may reduce (assumed) limitations on the use of saliva, particularly for diagnostic applications.

## Data Availability

The processed cortisol and ion concentration data has been published and can be accessed through the following DOI: https:/doi.org/10.48338/VU01-5ABDA3. The data is available upon request for reuse.
